# From Sample to Sequencing: The Importance of Pre-Analytical Sample Treatment in NGS Analysis of Patients with Chronic Lymphocytic Leukemia

**DOI:** 10.3390/cancers17223668

**Published:** 2025-11-15

**Authors:** Mirjana Suver Stević, Hrvoje Holik, Vlatka Periša, Saška Marczi, Nikolina Kolobarić, Marina Samardžija

**Affiliations:** 1 Clinical Institute for Transfusion Medicine, University Hospital Center Osijek, HR-31000 Osijek, Croatia; perisa.vlatka@kbco.hr (V.P.); marczi.saska@kbco.hr (S.M.); samardzija.marina@kbco.hr (M.S.); 2 Faculty of Medicine, University Josip Juraj Strossmayer of Osijek, HR-31000 Osijek, Croatia; 3 Department of Biology, University Josip Juraj Strossmayer of Osijek, HR-31000 Osijek, Croatia; 4 General Hospital Dr. Josip Benčević, Slavonski Brod, HR-35000 Slavonski Brod, Croatia; hematologija@bolnicasb.hr

**Keywords:** genetic testing, leukemia, lymphocytic, leukocytes, mononuclear, B-lymphocytes, next-generation sequencing, *TP53*

## Abstract

Background/Objectives: Chronic lymphocytic leukemia (CLL) is a hematologic malignancy characterized by uncontrolled accumulation of B lymphocytes. A key feature of CLL is the presence of genetic aberrations, particularly alterations of chromosome 17, such as deletion of 17q and/or mutations in the *TP53* gene. Since these abnormalities are highly relevant for therapeutic decision-making, assessment of *TP53* mutational status is strongly recommended in routine diagnostics. This study aimed to evaluate the reliability of *TP53* sequencing results depending on the type of DNA sample analyzed. Methods: DNA was isolated from two different sample types of the same patient: mononuclear cells (CLL1) and purified CD19+ cells (CLL2). The entire coding region of *TP53* (exons 2–11), including splice sites (+/− 10 bp), was analyzed using capture-based next-generation sequencing (NGS). Reads were aligned to the GRCh37/hg19 reference genome, and variants were interpreted using DRAGEN Enrichment (Illumina) and Franklin (QIAGEN). Results: In sample CLL1, the NM_000546.6:c.626_627del mutation (Tier I) was identified with a variant allele frequency (VAF) of 57.06%. The same mutation was confirmed in CLL2, but with a higher VAF of 94.78%. Importantly, an additional Tier I mutation (NM_000546.6:c.825_826del) was detected exclusively in CLL2 at a VAF of 1.59%. Both findings met the required sequencing depth as well as coverage per sample, confirming their validity. Conclusions: The study demonstrates that inadequate starting material for DNA isolation may mask low-frequency *TP53* mutations, resulting in false-negative results. Accurate detection requires ensuring sufficient CD19+ cell content, which is critical for reliable diagnostics and supports personalized treatment approaches in CLL.

## Simple Summary

Chronic lymphocytic leukemia (CLL) is a hematologic malignancy characterized by uncontrolled accumulation of B lymphocytes, frequently associated with genetic abnormalities. Among these, alterations of chromosome 17 and *TP53* mutations are the most clinically relevant for therapeutic decision-making. Therefore, assessment of *TP53* mutational status is strongly recommended in routine diagnostics. This study evaluated the reliability of sequencing results depending on the type of sample used for DNA isolation. Two samples from the same patient were analyzed: DNA from mononuclear cells and DNA from purified CD19+ cells. Next-generation sequencing (NGS) revealed the c.626_627del mutation in both samples, while an additional mutation (c.825_826del) was detected only in sample of DNA isolated from CD19+ cells. These findings highlight that inappropriate starting material may lead to false-negative results, particularly when mutation frequency is low. Ensuring sufficient CD19+ cell content prior to sequencing is essential for accurate detection, enabling precise diagnostics and supporting personalized therapy in CLL patients.

## 1. Introduction

Chronic lymphocytic leukemia (CLL) is the most common type of leukemia in adults in the Western world. It is characterized by an uncontrolled accumulation of monoclonal B cells [1,2]. Due to genetic alterations, the transformed B cells lose their normal physiological functions and evade programmed cell death, which enables their long-term survival. The development of CLL results from the interplay of genetic, environmental, and immunological factors. Age-related somatic mutations and defective DNA repair, combined with chronic B cell receptor stimulation, promote malignant transformation of B cells. Familial predisposition and environmental exposures further contribute to genomic instability and disease onset. Characteristic features of CLL include various chromosomal aberrations such as trisomy 12, as well as deletions of the long arm del(q) of chromosomes 11, 13, and short arm del(p) of chromosome 17, in addition to somatic mutations in *TP53* located on chromosome 17p. There are no specific clinical symptoms indicating the presence of *TP53* aberrations; rather, nonspecific manifestations such as night sweats, fever, weight loss, fatigue, recurrent infections, anemia, and bleeding reflect disease activity and may suggest a new diagnosis or relapse of CLL [3,4,5,6]. These aberrations are associated with a poor prognosis and resistance to standard chemoimmunotherapy (CIT) [7,8]. According to current data, approximately 80% of patients harboring del(17p) also carry *TP53* mutations in the second allele [9]. *TP53* aberrations are considered among the strongest prognostic and predictive markers for treatment decisions in CLL [10]. In patients with a confirmed del(17p) or a clinically significant *TP53* mutation, the treatment options are therefore Bruton’s tyrosine kinase or BCL2 inhibitors [11]. The determination of *TP53* aberration status is therefore a key factor in the choice of therapy and disease prognosis.

The *TP53* gene consists of 11 exons, with most mutations occurring in the DNA binding domain encoded by exons 4–11. The highest proportion of mutations is found in exons 5 and 7, followed by exons 8, 6, and 4. Regarding the type of mutation is concerned, more than 70% are missense mutations. At the time of diagnosis, only a small proportion of patients (around 5–10%) have some of the clinically important *TP53* aberrations. The frequency of these mutations increases over time and with therapy. Of these, less than 10% have only del(17p) without mutations on the other allele. A high variant allele frequency (VAF) was found in 60% of patients, and in this group the ratio of patients with and without del(17p) was similar. In addition, in patients with low VAF, defined as a mutant allele frequency below 10%, a much lower proportion also had del(17p) on the other allele. The clinical relevance of mutations with low VAF is still under discussion.

Given the pivotal role of chromosomal aberrations in disease biology, the assessment of the 17q aberration status, and, in particular, the mutational status of *TP53*, has become an indispensable component of clinical diagnostics. The European Research Initiative on CLL (ERIC) recommends routine testing of *TP53* mutations prior to treatment initiation, as well as at each relapse or before any change in therapy [12,13]. Reliable detection of *TP53* mutations is typically achieved by Sanger sequencing, with a sensitivity threshold above 10%, or by next-generation sequencing (NGS), which, due to its high throughput, enables the detection of low-frequency variants present at levels around 1%.

Accurate and reproducible results are essential for guiding therapeutic strategies in a personalized medicine context. Therefore, before implementing a molecular method into diagnostic practice, thorough method validation is required, including the assessment of result reproducibility, which is confirmed by repeated analyses of the same samples, as performed in this study. Such reliability depends not only on the quality of the biological sample but also on standardized laboratory procedures that minimize the risk of false-positive or false-negative findings. This includes the selection of appropriate methods and reagents, as well as the application of robust bioinformatic pipelines for secondary and tertiary data analysis.

Equally important is the rigorous interpretation and standardized reporting of results. To this end, compliance with international guidelines and nomenclature rules, such as those established by the Human Genome Variation Society (HGVS) [10], is mandatory to ensure unambiguous clinical communication. Adherence to these principles across all stages of the workflow ultimately provides the accuracy and confidence required for the implementation of reliable personalized medicine approaches.

## 2. Materials and Methods

The objective of this study was to perform a molecular analysis of a sample from a patient with CLL using NGS, with the aim of evaluating two distinct approaches to sample processing prior to DNA isolation. The implementation of the entire workflow for sample processing, NGS and result analysis is conducted in accordance with the recommendations of the ERIC consortium [12,13]. Investigation was approved by the Ethical Committee of the University Hospital Center Osijek, Osijek, Croatia, and performed in accordance with the Declaration of Helsinki and the Principles of Good Laboratory Practice, as well as Croatian law governing healthcare and the Law on the Protection of Patients’ Rights.

### 2.1. Isolation of Mononuclear Cells and Determination of CLL B Lymphocyte Percentage

Peripheral blood samples were collected into two K_2_EDTA-containing vacutainers and mononuclear cells were subsequently separated using a density gradient with Lymphodex™ (inno-train Diagnostik GmbH, Kronberg, Germany). To determine the proportion of CLL B lymphocytes, the isolated cells were incubated with BD CD5 FITC and BD CD19 APC antibodies (BD Bioscience, Sparks, MD, USA). Cells were incubated for 20 min at room temperature in the dark, washed with 1× PBS (Lonza, Verviers, Belgium), and resuspended in 350 µL 1× PBS. Flow cytometric analysis was performed using a BD FACSCanto™ II Clinical Flow Cytometry System (BD Bioscience, Sparks, MD, USA) to quantify the percentage of B-CLL lymphocytes.

### 2.2. Isolation of CD19+ Cells

CD19+ cells were isolated from mononuclear cell sample by positive selection using Dynabeads™ CD19 Pan B magnetic beads (Invitrogen™ by Thermo Fisher Scientific, Vilnius, Lithuania). Prior to use, the magnetic beads were thoroughly resuspended and washed with 1× PBS. The mononuclear cell suspension was added to the beads and incubated for 20 min at 4 °C. Following incubation, the tube was placed on a magnetic stand for 2 min, and the supernatant was discarded. The bead-bound cells were washed three times with 1× PBS and finally resuspended in 250 µL 1× PBS.

### 2.3. DNA Extraction

DNA was extracted from both, the mononuclear cell population (CLL1) and the CD19+ cell fraction (CLL2), using the QIAamp^®^ DNA Blood Mini Kit (Qiagen, Hilden, Germany), according to manufacturer’s instructions. Forty microliters of proteinase K and 400 µL of AL buffer were added to each sample, followed by vigorous vortexing for 1 min, and incubation at 56 °C for 10 min. For the CD19+ fraction, which remained bound to magnetic beads, the sample was placed on a magnetic stand for 5 min, and the supernatant was carefully transferred to a new 1.5 mL tube. Subsequently, 400 µL of 96% ethanol was added in both samples, and the mixtures were vortexed before transferring to a spin column. Both samples were centrifuged simultaneously at 14,000 rpm for 1 min. The columns were washed sequentially with AW1 and AW2 buffers and DNA was finally eluted using AE buffer, followed by centrifugation at 8000 rpm for 1 min.

### 2.4. Library Preparation and Sequencing

To determine the mutational status of *TP53* (NC_000017.10, NM_000546.5, LRG_321.t1), the entire coding region of the gene (exons 2–11) and splice sites including ±10 intronic nucleotides were analyzed. In addition to *TP53*, a custom panel (xGen™ Custom Panel, IDT, Coralville, IA, USA) was designed for the analysis of three additional genes relevant for therapy selection (*BCL2*, *BTK*, and *PLCG2*). Library preparation was performed using the Illumina™ DNA Prep with Enrichment workflow (Illumina, San Diego, CA, USA), following the manufacturer’s instructions. Protocol for NGS analysis involved multiple sequential steps: enzymatic DNA fragmentation, purification of generated fragments, sample indexing and fragment amplification, library purification, capture of target genomic region using capture probes, hybridization with biotinylated probes, additional amplification and purification of enriched library, followed by sequencing. The starting DNA input for both samples was 700 ng. Both samples were analyzed in the same sequencing run. To ensure sequencing quality control, a PhiX Control v3 (Illumina, San Diego, CA, USA) was included in the analysis. Prepared libraries were sequenced using the MiniSeq™ Mid Output Kit (300 Cycles) on the Illumina™ MiniSeq instrument (Illumina, San Diego, CA, USA). The method’s limit of detection (LOD) for variant allele frequency (VAF) was 1%, with a minimum mutation sequencing read depth of >750 and minimal coverage per sample >100,000 reads. For a result to be considered valid, each mutated variant must be supported by a minimum of 10 reads.

### 2.5. Bioinformatic Data Processing and Variant Interpretation

Nucleotide sequences were aligned to the human reference genome GRCh39/hg17, after which secondary and tertiary analyses were performed using the online platforms DRAGEN Enrichment (Illumina™, San Diego, CA, USA) and Franklin (Qiagen, Hilden, Germany). Franklin (QIAGEN) is a cloud-based bioinformatics platform designed for the analysis and interpretation of NGS data and integrates variant calling, annotation, and classification with curated databases, providing standardized and reproducible results suitable for clinical diagnostics. Verification of nomenclature and generation of complete *TP53* variant descriptions in accordance with HGVS and GRCh37/hg19 standards, including mutation classification, genomic position (exonic or intronic), frequency of occurrence in the *TP53* database, and full annotation according to the Locus Reference Genomic (LRG) framework, were carried out using the online tools Seshat [14] and LUMC Mutalyzer 3 version 3.1.1. Aligned sequencing reads in .bam files, along with variant calls in .vcf files, were analyzed and visualized using the Integrative Genomics Viewer (IGV) [15,16]. Interpretation and reporting of identified variants were performed in compliance with HGVS nomenclature, including reference sequence, transcript and protein, as well as pathogenicity class, clinical significance (Tier), and VAF.

Correlation analysis among the samples was performed using Pearson’s correlation test (MedCalc Software Ltd., https://www.medcalc.org/en/calc/test_correlation.php (Version 23.4; accessed on 10 November 2025).

## 3. Results

Peripheral blood samples were obtained from an 81-year-old male undergoing evaluation for suspected CLL. Laboratory analysis demonstrated leukocytosis with lymphocytosis (white blood cell counts 51.56 × 10^9^/L; absolute lymphocyte counts 40.51 × 10^9^/L) and normocytic anemia (erythrocyte count 3.57 × 10^12^/L; hemoglobin 104 g/L; mean corpuscular volume 90.8 fL). FISH analysis revealed biallelic deletion of the *Rb1* gene in 86% of interphase nuclei. No deletions of 11q, 17p and 13q34, as well as trisomy of chromosome 12 were detected.

Flow cytometry revealed that the population of B-CLL cells in the peripheral blood sample accounted for 53%. Following NGS analysis, both tested samples met the quality parameters confirming the reliability and robustness of the obtained results (Table 1).

After the secondary analysis, a tertiary analysis was performed using the Franklin online software, v.83 (Qiagen, Hilden, Germany). The obtained data revealed differences in the identified variants among the compared samples (Table 2). Specifically, in both analyzed samples the same mutated variant was detected, c.626_627del,p.(Arg209LysfsTer6). Mutation is located in exon 6 of the *TP53* gene. According to the hg19 genome reference, this mutation has a start position at 7,578,223 and end position at 7,572,222, representing a deletion of two nucleotides (GA) at the indicated genomic site.

In the CLL2 sample, the presence of the same mutation was confirmed; however, an additional mutation was also identified. The mutation c.825_826del,p.(Ala276fs*29), which is likewise categorized as pathogenic with clinical significance Tier I is located in exon 8 at genomic positions 7,577,113–7,577,112 and corresponds to a deletion of two nucleotides (TG). Both identified mutations are frameshift mutations that lead to the production of truncated and nonfunctional proteins.

The read count for the regions harboring variants in both samples exceeded 800, thereby meeting the required sequencing depth threshold of ≥750× to achieve a 1% sensitivity reporting level. Furthermore, since a mutated variant with VAF of 1.59% was detected in the CLL2 sample, visual inspection of the read alignment was performed using IGV software. The analysis confirmed that the mutation was not located at the edges of the sequenced fragments, excluding the possibility of a false-positive result. Correlation analysis of mutations c.626_627del and c.825_826del between CLL1 and CLL2 samples indicated a significant positive correlation relationship between VAF detection (Pearson’s correlation r = 0.99, *p* = 0.01, 95% CI 0.59–0.99)

To validate the detected mutation with a low VAF, the sample was reanalyzed, confirming an almost identical result (detection of mutation c.626_627del,p.(R209Kfs*6) VAF = 96.56% and mutation c.825_826del,p.(A276Lfs*29) VAF = 1.36%). The reproducibility of the method was assessed by comparing the results obtained from two independent replicates. For the low-frequency variant c.825_826del, the mean VAF was 1.47% with a standard deviation of 0.14 percentage points, corresponding to a coefficient of variation (%CV) of 9.62% and a relative percent difference (RPD) of 13.61%. For the high-frequency variant c.626_627del, the mean VAF was 95.72% with a standard deviation of 1.32, yielding a %CV of 1.38% and an RPD of 1.95%. These results demonstrate good reproducibility of the method, with higher variability observed only at low variant allele frequencies, which is expected near the limit of detection. In addition to the method validation performed before implementing the analysis in routine practice, this further confirmed the reproducibility of displayed results.

## 4. Discussion

The development and implementation of molecular diagnostic methods in clinical practice have significantly improved diagnostic approaches and enabled individualized treatment through targeted therapies. CLL represents one of the diseases where different therapeutic strategies can be applied. According to the ESMO guidelines [2,17], the diagnosis of CLL is based on several criteria, including the presence of monoclonal B lymphocytes in peripheral blood at concentrations exceeding 5 × 10^9^/L, with clonality confirmed by flow cytometry. Another criterion includes the presence of mature lymphocytes with a narrow rim of cytoplasm and dense nuclei without visible nucleoli, exhibiting partially aggregated chromatin.

In symptomatic patients, prior to initiating therapy, it is recommended to perform FISH analysis and molecular testing for *TP53* mutations if cytogenetic analysis does not reveal a 17p deletion. Since CLL is characterized by various genomic aberrations, including deletion of the short arm of chromosome 17 and/or *TP53* mutations, their presence or absence determines the treatment pathway, namely the choice between CIT and targeted therapies. Consequently, molecular testing of *TP53*, including sequencing, has become an essential part of diagnostic workup at both initial diagnosis and relapse [12,13].

Currently, *TP53* sequencing in clinical practice relies on Sanger sequencing and NGS. According to ERIC recommendations, both methods are valid for the molecular diagnosis of CLL, as they can reliably detect mutations with a VAF > 10%. Sanger sequencing has lower sensitivity (detection limit ≥ 10%) and allows the analysis of only a single gene or gene fragment per run. In contrast, NGS offers higher throughput and sensitivity, enabling the detection of variants with VAF ≤ 1% and the simultaneous analysis of multiple genes. In our laboratory, eight samples and four genes (*TP53*, *BLC2*, *BTK*, and *PLCG1*) are analyzed per run [18].

The increased sensitivity of NGS introduces concerns about possible false-positive and false-negative findings, as well as the clinical significance of mutations with a VAF below 10%. While enhanced sensitivity allows NGS to detect low-frequency variants, including those close to the detection threshold, it also increases the risk of false positives. These may arise from sequencing errors, PCR artifacts, or background noise inherent to the method. Accurate interpretation requires in-depth knowledge of NGS technology, bioinformatics pipelines, and thorough method validation. Lowering the VAF threshold increases the risk of misinterpreting background noise as true variants, leading to false-positive results. Conversely, high background noise, insufficient DNA input, low coverage, or poor sequencing quality may mask low-frequency variants, resulting in false negatives. To address this, our laboratory followed current international recommendations [17,19,20,21] using 700 ng of input DNA per sample, exceeding the minimal required amount and ensuring sufficient template for reliable variant detection.

Pre-analytical sample processing represents a crucial determinant of reliable molecular testing. According to ERIC guidelines, peripheral blood or bone marrow should be used as the starting material, whereas FFPE tissue is discouraged because of DNA fragmentation and degradation [12]. An additional important factor is the assessment of lymphocyte composition, specifically the proportion of CLL B cells. In line with ERIC recommendations and Croatian national criteria for NGS testing in CLL patients [12,13,18], a minimum lymphocyte fraction of 60–70% is required to ensure robust and interpretable sequencing results. However, samples that meet this threshold may still predominantly contain T cells, which are irrelevant for CLL analysis. Therefore, accurate determination of the CD19+ B-cell percentage is essential to avoid false-negative results. Our experience highlights this challenge. We frequently received samples with a high overall lymphocyte count, yet with <50% CLL B cells confirmed by flow cytometry. In such cases, the reliability of detecting low-VAF *TP53* variants becomes questionable. This was exemplified in our study by the variant c.825_826del (p.(A276Lfs*29)), detected at a VAF of 1.59%. Repeating the NGS analysis yielded an almost identical VAF value, thereby confirming both high- and low-VAF mutations and demonstrating assay reproducibility, a key requirement for clinical implementation [22,23]. Our results underscore the importance of appropriate sample selection for DNA extraction and *TP53* testing. A sample with 53% CLL B cells proved inadequate, as the low-burden pathogenic mutation (c.825_826del (p.(A276Lfs*29)) was not detected by NGS, whereas both low- and high-burden mutations were detected in the CD19+ fraction.

Our study is based on a single patient due to the complexity and cost of obtaining samples with both low- and high-VAF *TP53* mutations. Mutational status is assessed only at specific clinical time points, requiring larger blood volumes for isolation of mononuclear and CD19+ cells. Although longer disease duration and prior CIT may increase mutation likelihood, these are not reliable predictors. During method validation, four patients were analyzed, with each patient’s sample sequenced from both mononuclear cells and CD19+ cells (a total of eight samples per batch). *TP53* mutations—both high- and low-VAF—were detected in only one patient, highlighting the challenge of identifying samples with this specific mutational profile. To ensure consistency and reliability, our laboratory now uses isolated CD19+ B cells as the standard input material according to Guidelines for the Molecular-Diagnostic Procedure of NGS of Croatian Cooperative Group for Hematologic Disease [18].

Multiple factors can influence the final analytical outcome, so reports must clearly specify the sample type and methods used for cell isolation to ensure accurate interpretation and reliability. This is particularly important for measurable residual disease (MRD) monitoring, where results are only comparable if identical sample types are used consistently.

Current CLL diagnostic guidelines emphasize reporting low-VAF *TP53* mutations in clones/subclones that may expand during relapse and develop resistance to CIT [13]. The clinical significance of low VAF *TP53* mutations remains unclear, particularly regarding their impact on time to first treatment (TTFT) and overall survival (OS). Initially, only a small proportion of patients present with *TP53* aberrations. However, under treatment pressure—CIT in patients without *TP53* aberrations and targeted therapies in those with *TP53* aberrations—mutational status and/or clonal composition can evolve [9,24]. The 2019 ERIC guidelines recommended reporting only *TP53* mutations with VAF ≥ 10%, matching Sanger’s detection threshold [12]. Low VAF variants were not mandatory to report due to uncertain clinical significance, possible technical artifacts, and variability between NGS platforms and bioinformatic analyses. Recent evidence, however, highlights the potential clinical relevance of low VAF *TP53* mutations, as these subclones may confer resistance to CIT and expand during relapse [24,25,26,27]. Most data come from retrospective studies comparing progression-free survival (PFS) and OS [28,29,30,31,32]. Although results are not entirely consistent, most suggest shorter survival in patients harboring low-burden *TP53* mutations. Accordingly, the updated 2024 ERIC guidelines [13] recommend reporting all clinically relevant Tier I–III variants (pathogenic, likely pathogenic, or of uncertain significance) regardless of VAF, aligning with treatment recommendations to avoid CIT in any patient with *TP53* aberrations. With regard to the treatment of CLL patients, the choice of therapy depends on the VAF percentage of *TP53* mutations. Patients with del(17p) or *TP53* mutations represent a distinct high-risk category. In these patients, CIT should be avoided. It is recommended that they receive continuous BTK inhibitor therapy, either alone or in combination with venetoclax, as these agents have demonstrated durable long-term disease control [33,34]. Our findings support a nuanced therapeutic approach to *TP53*-mutated CLL based on clonal composition. Patients harboring *TP53* mutations (detectable in standard CLL1 analysis with high VAF) may be eligible for fixed-duration BTK inhibitor therapy combined with venetoclax, followed by treatment-free intervals and reinduction at the time of disease progression. In contrast, patients with subclonal *TP53* mutations detected exclusively through CD19-enriched NGS analysis (CLL2) should receive continuous BTK inhibitor monotherapy without a predetermined cessation endpoint, as subclonal *TP53* aberrations confer a similarly poor prognosis and resistance pattern as dominant clones. This stratification ensures that patients with cryptic high-risk genetics (low-VAF *TP53*) receive appropriately intensive therapy from the outset, preventing inadequate initial treatment and subsequent therapy failure.

In conclusion, pre-analytical sample processing is equally important as other analytical steps, including library preparation, sequencing, and data analysis. It should be standardized and never underestimated to ensure accurate detection of both high- and low-frequency *TP53* mutations in CLL.

## 5. Conclusions

This study underscores the pivotal role of rigorous pre-analytical sample processing, as it exerts a direct impact on the accuracy and reliability of molecular diagnostic outcomes. Inadequate specimen preparation—most notably the inappropriate selection of the initial material for DNA extraction—can result in false-negative findings, particularly in cases where the relevant mutation is confined to a small fraction of cells. Such diagnostic discrepancies may profoundly influence therapeutic decision-making and the overall course of patient management. Accordingly, a comprehensive understanding of both disease biology and the methodological scope and limitations of molecular diagnostic procedures is indispensable. Only through such an integrated approach to sample handling and analysis can robust, reliable, and clinically actionable results be ensured.

## Figures and Tables

**Table 1:** Overview of parameters and values recorded during NGS analysis and secondary data processing using the DRAGEN Enrichment software v.4.4.6. (Illumina, San Diego, CA, USA).

Parameter	CLL1 *	CLL2 ^+^
Total aligned reads per sample ^#^	4,919,644	5,469,312
Percent Q30 Bases	91.89%	90.78%
Mean Target Coverage Depth	2117.4	2533.9
Uniformity of Coverage (Pct > 0.2 mean)	98.53%	98.71%

* DNA isolated from mononuclear cell fraction; ^+^ DNA isolated from the CD19+ cell population; ^#^ According to the ERIC guidelines, a reliable result requires a minimum coverage of 100,000 reads per sample.

**Table 2:** Tertiary analysis results using Franklin online software (by Qiagen, Hilden, Germany): variant allele frequency and read depth.

	Sample			
	CLL1		CLL2	
	VAF (%)	Number of Variant Reads/Total Number of Sequencing Reads at Specific Nucleotide Position	VAF (%)	Number of Variant Reads/Total Number of Sequencing Reads at Specific Nucleotide Position
c.626_627del,p.(R209Kfs*6)	57.06	489/857	94.78	836/882
c.825_826del,p.(A276Lfs*29)	-	-	1.59	14/881

Variant Allele Frequency (VAF): proportion of sequencing reads supporting the variant relative to the total reads at that genomic position. Read depth: total number of sequencing reads covering the specific nucleotide position. CLL1–sample of DNA extracted from separated mononuclear cells using density gradient with Lymphodex™; CLL2–sample of DNA extracted from CD19+ cell population after mononuclear cells separation method.

## Data Availability

The original contributions presented in this study are included in the article. Further inquiries can be directed to the corresponding author.
